# Identification and Characterization of Two Regiospecific Tricetin UDP-Dependent Glycosyltransferases from Pomegranate (*Punica granatum* L.)

**DOI:** 10.3390/plants11060810

**Published:** 2022-03-18

**Authors:** Sheng Wu, Lijing Chang, Li Tian

**Affiliations:** 1 Shanghai Key Laboratory of Plant Functional Genomics and Resources, Shanghai Chenshan Botanical Garden, Shanghai 201602, China; wusheng@sioc.ac.cn (S.W.); changlijing@csnbgsh.cn (L.C.); 2 Shanghai Chenshan Plant Science Research Center, Chinese Academy of Sciences, Shanghai 201602, China; 3 Department of Plant Sciences, University of California, Davis, CA 95616, USA

**Keywords:** anther, petal, tricetin, tricetin 4′-*O*-glucoside, 4′-*O*-UGT, pomegranate

## Abstract

Tricetin (5,7,3′,4′,5′-pentahydroxyflavone) is a dietary flavone from flowers of Myrtales plants with demonstrated functions in promoting human health. By contrast, the bioactivity of its glucosylated derivative tricetin 4′-*O*-glucoside has not been extensively explored. We conducted metabolite profiling analysis of pomegranate (a Myrtales plant) floral tissues and revealed that tricetin and tricetin 4′-*O*-glucoside accumulate in anthers, but not petals. In addition, the comparative analysis of anther and petal transcriptomes identified 10 *UGTs* that are more highly expressed in anthers than petals. Of the 10 UGTs, *Pg*UGT76Z1 and *Pg*UGT73AL1 glucosylated specifically at the 4′-*O* position of tricetin to form tricetin 4′-*O*-glucoside. The phylogenetic analysis indicated that *Pg*UGT76Z1 and *Pg*UGT73AL1 belong to different plant UGT groups, suggesting a convergent evolution of these tricetin UGTs. Overall, identification and characterization of *Pg*UGT76Z1 and *Pg*UGT73AL1 not only provides evolutionary insights into tricetin glucosylation, but also offers an opportunity to produce tricetin 4′-*O*-glucoside in large quantities through microbial biotransformation or plant metabolic engineering, thus facilitating the investigation of tricetin 4′-*O*-glucoside bioactivities.

## 1. Introduction

Pomegranate (*Punica granatum* L.) is an edible medicinal plant in the order of Myrtales. Many parts of pomegranate plants, such as flowers, fruits, and leaves, have been used for medicinal purposes, largely due to the abundant hydrolyzable tannins (HTs; including ellagitannins and gallotannins) and flavonoids present in these tissues [1]. However, most studies have mainly focused on analyzing the metabolite profiles in pomegranate fruits, with those in other tissues relatively underexplored. Recently, two flavonoid compounds, tricetin (5,7,3′,4′,5′-pentahydroxyflavone) and tricetin 4′-*O*-glucoside, were identified from pomegranate flowers using mass spectrometry (MS) and nuclear magnetic resonance (NMR) analyses [2]. Tricetin was shown to possess anticancer (breast, liver, and lung cancers) and anti-inflammatory activities in studies using human cell lines [3,4,5,6]. In addition, anti-diabetic and anti-obesity activities in in vitro assays were also reported for tricetin [2]. Attachment of a glucose moiety to tricetin presumably changes the solubility and reactivity of tricetin 4′-*O*-glucoside, although its bioactivity has not been extensively studied.

To further examine metabolite accumulation in different pomegranate floral tissues, high-performance liquid chromatography (HPLC) analysis was carried out, indicating that tricetin is present in both anthers and filaments, whereas tricetin 4′-*O*-glucoside accumulates specifically in anthers of pomegranate flowers [7]. Glucosylation of tricetin to form tricetin 4′-*O*-glucoside is catalyzed by UDP-dependent glycosyltransferases (UGTs). A recently identified UGT in pomegranate, *Pg*UGT95B2, displayed a strong specific activity towards tricetin, but produced seven glucosylated products, suggesting that *Pg*UGT95B2 can glucosylate multiple hydroxyl groups of tricetin and likely generate tricetin derivatives with multiple glucosidic linkages [7]. However, it is unclear as to whether there is a UGT activity in pomegranate that can glucosylate specifically at the 4′-*O* position of tricetin.

To better understand the metabolite profiles of different floral tissues of pomegranate, anthers and petals were collected and analyzed using liquid chromatography high-resolution electrospray ionization mass spectrometry (LC-HR-ESI-MS) in this study. In addition to the unique presence of tricetin and tricetin 4′-*O*-glucoside in anthers, more flavonoid and HT compounds were also identified in anthers relative to petals. To identify UGTs that can regiospecifically modify tricetin and form tricetin 4′-*O*-glucoside, a comparative transcriptome analysis was carried out using anthers and petals of pomegranate flowers. Ten UGTs that were more highly expressed in anthers than petals were cloned and biochemically characterized. Two of the UGTs, *Pg*UGT73AL1 and *Pg*UGT76Z1, demonstrated glucosylation activities specific to the 4′-*O* position of tricetin.

## 2. Results

### 2.1. Metabolite Analysis Identified the Unique Accumulation of Tricetin and Tricetin 4′-O-glucoside, and Abundant Accumulation of Flavonoids and HTs in Anthers of Pomegranate Flowers

**Figure 1:**
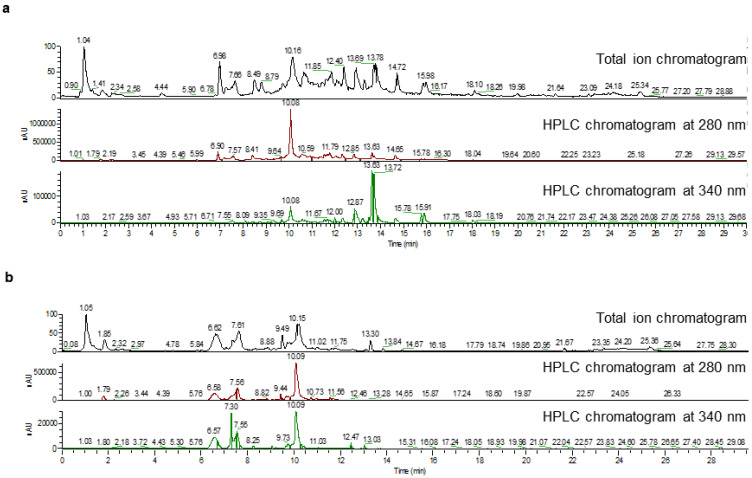
Metabolite analysis of pomegranate anthers and petals. (**a**) Chromatograms of liquid chromatography high-resolution electrospray ionization mass spectrometry (LC-HR-ESI-MS) for metabolites extracted from pomegranate anthers. (**b**) Chromatograms of LC-HR-ESI-MS for metabolites extracted from pomegranate petals.

To examine the compounds that accumulate in anthers and petals of pomegranate flowers, metabolites were extracted from both tissues and subjected to LC-HR-ESI-MS analysis (Figure 1; Tables S1 and S2). In contrast to the previous HPLC analysis of metabolites in flowers using several available standards for compound identification [7], the LC-HR-ESI-MS analysis obtains high-resolution MS data of the metabolites and allows tentative compound identification by comparing the retention times, molecular ions, and MS/MS fragment ions to those accessible in public databases. This approach enables identification of a larger collection of metabolites, as authentic standards are not available for many of them. A total of 80 and 52 metabolites were tentatively identified from anthers and petals, respectively (Tables S1 and S2). For HTs, multiple hexahydroxydiphenoyl (HHDP) glucoside derivatives, bis-HHDP glucoside derivatives, and galloyl-hexoside derivatives, as well as gallic acid, galloyl glucoside, tri-galloyl-glucose, tetra-galloyl-glucose, granatin A, granatin B, pedunculagin II, strictinin (an ellagitannin), and punigluconin were identified in both anthers and petals (Tables S1 and S2). The following HTs were identified in anthers but not petals: casuarinin, digalloyl glucoside, castalagin, punicafolin, ellagic acid, galloyl-bis-HHDP-hexoside, and pentagalloyl glucose (Tables S1 and S2).

For flavonoids, rutin, flavonoid glycoside, eriodictyol, hovetrichoside C (maesopsin 4-*O*-glucoside; an aurone glucoside), cyanidin 3-*O*-rutinoside, and cyanidin 3,5-di-*O*-glucoside were identified in both anthers and petals (Tables S1 and S2). The flavonoids that were specifically identified in petals include kaempferol 3-*O*-glucoside, apigenin 4-*O*-glucoside, and phlorizin. Those were only identified in anthers but not petals include luteolin 7-*O*-glucoside/kaempferol 4′-*O*-glucoside, kaempferol 7-*O*-glucoside, luteolin 3′-*O*-xylopyanoside, naringenin, isorhamnetin, luteolin, apigenin, phloretin, tricetin, and tricetin 4′-*O*-glucoside (Tables S1 and S2).

### 2.2. Comparative Transcriptome Analysis Coupled with Enzyme Activity Assays Identified Two Regiospecific UGTs for Tricetin 4′-O-glucoside Biosynthesis

Because tricetin and tricetin 4′-*O*-glucoside accumulate in anthers but not petals of pomegranate flowers, we hypothesized that gene(s) encoding UGT(s) responsible for tricetin 4′-*O*-glucoside biosynthesis could be more highly expressed in anthers than petals. To identify the UGT(s) that catalyze 4′-*O*-glucosylation of tricetin, comparative transcriptome analysis was carried out using anther and petal tissues of pomegranate (each with three biological replicates). Between 42 and 46 million raw 2 × 150 bp paired-end sequence reads were obtained for each transcriptome with Q30 values ranging from 88.8% to 92.7% (Table S3). For all transcriptomes, more than 87% of the cleaned sequence reads were mapped to the reference pomegranate genome [8] (Table S4). There are 236 genes that are more abundantly expressed in petals and 1359 genes more abundantly expressed in anthers according to the criteria of Log_2_ foldchange > 1 and an adjusted *p* < 0.05 (Tables S5 and S6). Consistent with the results from the metabolite analysis, the Kyoto Encyclopedia of Genes and Genomes (KEGG) pathway enrichment analysis showed that genes involved in flavonoid biosynthesis were enriched in the differentially expressed genes (DEGs) (Figure S1).

**Figure 2:**
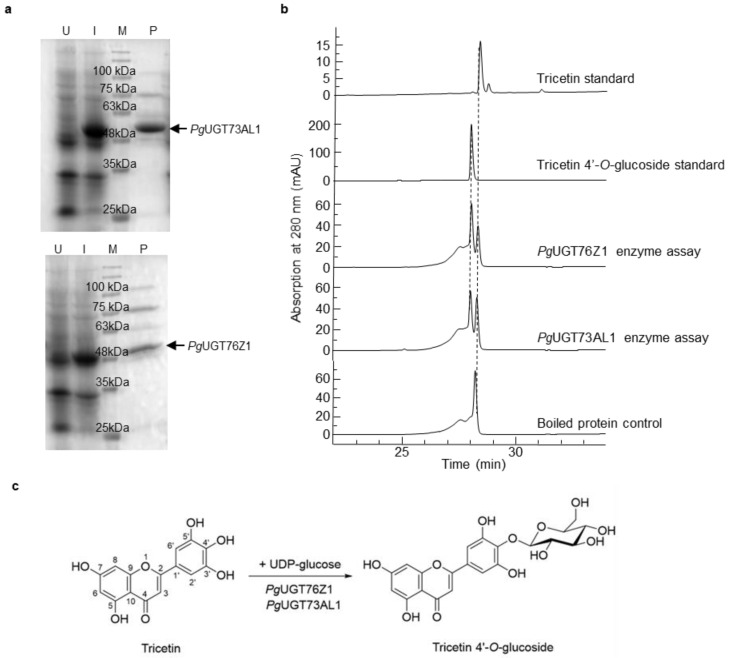
UGT protein expression and enzyme assays. (**a**) Expression and purification of *Pg*UGT73AL1 and *Pg*UGT76Z1 proteins. U, total lysate from uninduced *E. coli* cells transformed with the *PgUGT73AL1* or *PgUGT76Z1* plasmid construct; I, total lysate from *E. coli* cells transformed with the *PgUGT73AL1* or *PgUGT76Z1* plasmid construct and induced with 0.1 mM isopropyl β-D-1-thiogalactopyranoside; M, protein molecular mass marker; P, purified recombinant protein; kDa, kilodalton. (**b**) UGT enzyme activity assays with boiled UGT proteins (control) or purified recombinant *Pg*UGT73AL1 and *Pg*UGT76Z1 proteins. (**c**) Glucosylation of tricetin by *Pg*UGT73AL1 and *Pg*UGT76Z1 to form tricetin 4′-*O*-glucoside.

Among the more abundantly expressed genes in anthers relative to petals, 10 were annotated as *UGTs* (Table 1). These candidate *UGTs* were cloned and expressed as His-tagged recombinant proteins in *E. coli* (Figure 2a). Enzyme activity assays using purified recombinant UGTs showed that only *Pg*UGT73AL1 and *Pg*UGT76Z1 were able to use tricetin and UDP-glucose as substrates, and both reactions led to a single product tricetin 4′-*O*-glucoside (Figure 2b,c). No product was formed in the control reaction with boiled UGT proteins (Figure 2b). The analysis of *Pg*UGT73AL1 and *Pg*UGT76Z1 proteins using the TargetP 2.0 server did not identify any sorting signals for subcellular localization (data not shown), suggesting that both UGTs are located in the cytosol.

### 2.3. PgUGT73AL1 and PgUGT76Z1 Belong to Different Plant UGT Phylogenetic Groups

**Figure 3:**
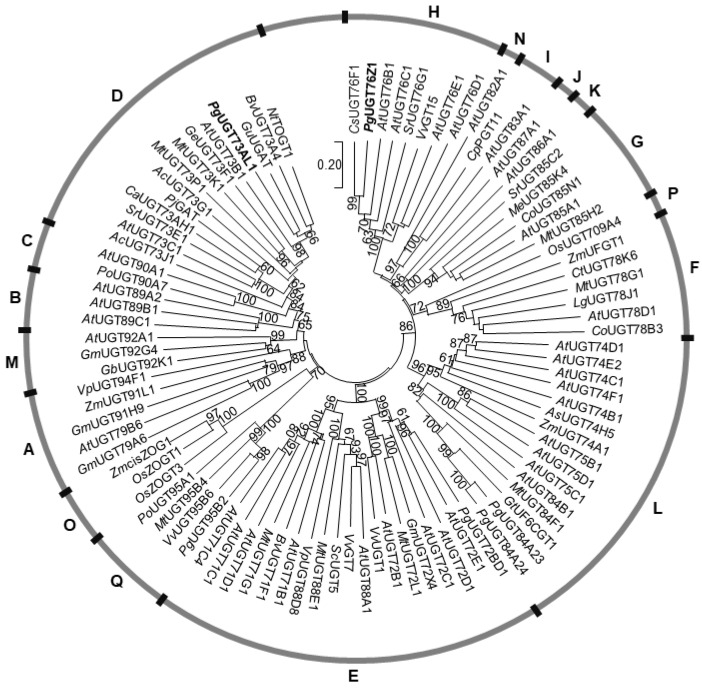
Phylogenetic analysis of *Pg*UGT73AL1, *Pg*UGT76Z1, and selected plant UGTs in phylogenetic groups A-Q. The phylogenetic tree was built using the neighbor-joining method and tested with 1000 replicates. *Pg*UGT73AL1 and *Pg*UGT76Z1 are highlighted in bold. Bootstrap values greater than 60 are shown.

To understand the evolutionary relationship of *Pg*UGT73AL1 and *Pg*UGT76Z1 with UGTs in pomegranate and other plants, a phylogenetic tree of selected functionally characterized plant UGTs was constructed using the neighbor-joining method (Figure 3). Of the 17 UGT phylogenetic groups (A–Q) delineated in plants, *Pg*UGT73AL1 and *Pg*UGT76Z1 belong to groups D and H, respectively, suggesting that they have evolved independently (Figure 3). Within the group D UGTs, *Pg*UGT73AL1 clustered closely with *Nt*TOGT1, which glucosylates scopoletin (a hydroxycoumarin) to produce scopolin [9]; *Bv*UGT73A4, which glucosylates a wide range of flavonoids to form flavonoid glucosides [10]; and *Gu*UGAT, which adds glucuronosyl groups to glycyrrhetinic acid (a triterpene derivative) to yield glycyrrhizin [11] (Figure 3). Within the group H UGTs, *Pg*UGT76Z1 was most closely clustered with *Cs*UGT76F1, which has shown flavonoid 7-*O*-glucosyltransferase and 7-*O*-rhamnosyltransferase activities [12] (Figure 3).

## 3. Discussion

In this study, we determined that both tricetin and tricetin 4′-*O*-glucoside accumulate in anthers but are absent in petals of pomegranate flowers (Tables S1 and S2). In addition, we identified 10 UGTs from the comparative transcriptome analysis that showed higher expression levels in anthers than petals (Table 1). Two of the candidate UGTs, *Pg*UGT73AL1 and *Pg*UGT76Z1, carried out regiospecific glucosylation of tricetin at the 4′-*O* position (Figure 2). *Pg*UGT73AL1 and *Pg*UGT76Z1 are predicted to be cytosolic proteins, suggesting that their substrate tricetin is also accessible in the cytosol. Tricetin could be produced in the cytosol or transported to the cytosol for the glucosylation reaction from another subcellular organelle where it is generated.

Glucosylation of tricetin is expected to change its hydrophobicity, and likely also its reactivity. In contrast to the multiple reports on the beneficial functions of tricetin to human health, the bioactivity of tricetin 4′-*O*-glucoside is underexplored. The regiospecific UGTs *Pg*UGT73AL1 and *Pg*UGT76Z1 can be expressed as recombinant proteins in microbes and used as biocatalysts for the synthesis of tricetin 4′-*O*-glucoside for drug discovery. Both *UGTs*, together with genes encoding enzymes for tricetin biosynthesis, can also be overexpressed in plants that do not naturally make tricetin and tricetin 4′-*O*-glucoside to produce these useful flavone compounds.

Besides applications in biotechnology, cloning and biochemical characterization of *Pg*UGT73AL1 and *Pg*UGT76Z1 also provide a glimpse into the evolution of UGTs that glucosylate tricetin. *Pg*UGT73AL1 and *Pg*UGT76Z1 are members of the plant UGT phylogenetic groups D and H, respectively (Figure 3). The previously characterized *Pg*UGT95B2 that glucosylates tricetin at multiple positions belongs to group Q [7]. The observation that UGTs in groups D, H, and Q can all glucosylate tricetin suggests the convergent evolution of these UGTs. It remains to be determined whether UGTs from other phylogenetic groups, besides those that were tested in this study, can also use tricetin as a substrate, and whether the glucosylation reaction is specific to the 4′-*O* position of tricetin.

Abundant HTs and flavonoids were identified in anthers and petals of pomegranate flowers, suggesting that they may play a role in these reproductive tissues (Tables S1 and S2). On the other hand, the unique accumulation of certain HTs and flavonoids in anthers or petals suggests that these compounds may function in a tissue-specific manner in pomegranate flowers. The transcriptome data could be further explored to identify the biosynthetic and/or catabolic genes for HTs and flavonoids that may account for the distinctive metabolite accumulation in these tissues. For example, an intriguing question remains to be answered: what is the mechanistic basis for the taxonomically restricted production and accumulation of tricetin in pollens of Myrtales plants (including pomegranate) [13]? It is possible that a specific hydroxylase that converts luteolin (tetrahydroxy flavone) to tricetin (pentahydroxy flavone) is uniquely expressed and/or active in pollens of these plants? In the future, the transcriptome data from anthers (pollen-bearing structure where tricetin is present), in comparison to those from petals (where tricetin is absent), of pomegranate flowers can be investigated to identify the candidate hydroxylase for tricetin production.

## 4. Materials and Methods

### 4.1. Metabolite Analysis

Anther and petal tissues were collected from blooming flowers of pomegranate cv. Wonderful. Three biological replicates (each was pooled from many flowers) were harvested for each tissue, ground in liquid nitrogen to a fine powder, and kept at −80 °C for metabolite and transcriptome analyses. For metabolite analysis, the ground pomegranate floral tissues were freeze-dried, and 50 mg of the lyophilized tissue was extracted with 1 mL of 70% methanol under sonication. After centrifugation at 13,000 rpm for 10 min, the supernatant of the extract was passed through a syringe filter (MilliporeSigma, Burlington, MA, USA) and subjected to LC-HR-ESI-MS analysis on an ultra-performance liquid chromatography (UPLC) (Waters, Milford, MA, USA) coupled to a Q Exactive mass spectrometer (Thermo Scientific, Waltham, MA, USA). The mass spectra from *m*/*z* 120 to 1800 were obtained in the positive (ion spray voltage/ISV of 4 kV) and negative (ISV of 3 kV) ion modes. The parameters for the LC-HR-ESI-MS analysis and metabolite identification using publicly available MS libraries were as previously described [7,14].

### 4.2. Transcriptome Analysis

For transcriptome analysis, total RNA was extracted from the ground anther and petal tissues using the Trizol reagent (Invitrogen, Carlsbad, CA, USA). After the quality control of total RNA using agarose gel electrophoresis and the Agilent 2100 Bioanalyzer (Agilent, Santa Clara, CA, USA), mRNA was enriched using the oligo (dT) magnetic beads. RNAseq libraries were constructed from mRNA samples using the Illumina TruSeq RNA sample prep kit (Illumina, San Diego, CA, USA) and sequenced on an Illumina HiSeq4000 instrument (Illumina).

The raw reads were cleaned by removing the 3′-end adapter and low-quality sequences using Cutadapt (https://cutadapt.readthedocs.io/en/stable/, accessed on 30 July 2018). For mapping of sequence reads, the reference pomegranate genome (ASM220158v1; GenBank accession number GCA_002201585.1) was downloaded from National Center for Biotechnology Information (NCBI). The trimmed reads were mapped to the reference pomegranate genome using Bowtie2 (v2.2.3; http://bowtie-bio.sourceforge.net, accessed on 18 November 2021) and Tophat2 (v2.1.1; https://ccb.jhu.edu/software/tophat/index.shtml, accessed on 18 November 2021) [15]. The read counts were quantified using HTSeq [16], and the reads per kilo bases per million reads (RPKM) values were calculated using RSeQC [17]. Differential gene expression analysis between anthers and petals was performed using DESeq2, with an adjusted *p*-value < 0.05 and |log_2_ FC| > 1 [18]. The KEGG pathway enrichment analysis was conducted by mapping DEGs to the KEGG pathways (http://www.genome.jp/kegg/, accessed on 18 November 2021) [19]. The transcriptome data were deposited in the NCBI sequence reads archive (SRA) under the accession number PRJNA806846.

### 4.3. Protein Sequence Analysis and Enzyme Assays

The open reading frames (ORFs) of the candidate *UGTs* were codon optimized for expression in *E. coli*, synthesized by Genewiz (Suzhou, China), and then cloned in the pET28a vector. The recombinant plasmids were transformed into *E. coli* BL21 (DE3) cells. Expression of UGT proteins was induced by adding isopropyl β-D-1-thiogalactopyranoside (IPTG) to a final concentration of 0.1 mM. Procedures for protein expression, purification of His-tagged recombinant proteins, UGT enzyme assays (0.25 mM tricetin was used in the assays), and HPLC analysis were as previously described [20]. The TargetP 2.0 server (https://services.healthtech.dtu.dk/service.php?TargetP-2.0, accessed on 16 December 2021) was used for prediction of subcellular sorting signals in protein sequences.

### 4.4. Phylogenetic Analysis

Protein sequences of *Pg*UGT76Z1 and *Pg*UGT73AL1, along with those of selected plant UGTs, were aligned using Multiple Sequence Comparison by Log-Expectation (MUSCLE) [21]. A neighbor-joining tree was constructed based on the protein sequence alignment using Molecular Evolutionary Genetics Analysis (MEGA) [22]. The Arabidopsis Genome Initiative (Arabidopsis sequences) and GenBank (non-Arabidopsis sequences) accession numbers for the UGTs are as follows: *Ac*UGT73G1 (AAP88406), *Ac*UGT73J1 (AAP88407), *As*UGT74H5 (ACD03250), *At*UGT71B1 (AT3G21750), *At*UGT71C1 (AT2G29750), *At*UGT71C4 (AT1G07250), *At*UGT71D1 (AT2G29730), *At*UGT72B1 (AT4G01070), *At*UGT72C1 (AT4G36770), *At*UGT72D1 (AT2G18570), *At*UGT72E1 (AT3G50740), *At*UGT73B1 (AT4G34138), *At*UGT73C1 (AT2G36750), *At*UGT74B1 (AT1G24100), *At*UGT74C1 (AT2G31790), *At*UGT74D1 (AT2G31750), *At*UGT74E2 (AT1G05680), *At*UGT74F1 (AT2G43840), *At*UGT75B1 (AT1G05560), *At*UGT75C1 (AT4G14090), *At*UGT75D1 (AT4G15550), *At*UGT76B1 (AT3G11340), *At*UGT76C1 (AT5G05870), *At*UGT76D1 (AT2G26480), *At*UGT76E1 (AT5G59580), *At*UGT78D1 (AT1G30530), *At*UGT79B6 (AT5G54010), *At*UGT82A1 (AT3G22250), *At*UGT83A1 (AT3G02100), *At*UGT84B1 (AT2G23260), *At*UGT85A1 (AT1G22400), *At*UGT86A1 (AT2G36970), *At*UGT87A1 (AT2G30150), *At*UGT88A1 (AT3G16520), *At*UGT89A2 (AT5G03490), *At*UGT89B1 (AT1G73880), *At*UGT89C1 (AT1G06000), *At*UGT90A1 (AT2G16890), *At*UGT92A1 (AT5G12890), *Bv*UGT71F1 (AY526081), *Bv*UGT73A4 (AY526080), *Ca*UGT73AH1 (AUR26623), *Co*UGT78B3 (AEB61484), *Co*UGT85N1 (AEB61489), *Cp*PGT11 (AIS39477), *Cs*UGT76F1 (KDO69246), *Ct*UGT78K6 (BAF49297), *Gb*UGT92K1 (ASK39406), *Ge*UGT73F1 (BAC78438), *Gm*UGT72 × 4 (KRH46505), *Gm*UGT79A6 (BAN91401), *Gm*UGT91H9 (NP_001348424), *Gm*UGT92G4 (KRH14708), *Gt*UF6CGT (AB985754), *Gu*UGAT (ANJ03631), *Lg*UGT78J1 (AEB61487), *Me*UGT85K4 (AEO45781), *Mt*UGT71G1 (AAW56092), *Mt*UGT72L1 (ACC38470), *Mt*UGT73K1 (AAW56091), *Mt*UGT73P1 (ABI94026), *Mt*UGT78G1 (ABI94025), *Mt*UGT84F1 (ABI94023), *Mt*UGT85H2 (ABI94024), *Mt*UGT88E1 (ABI94021), *Mt*UGT95B4 (XP_003612636), *Nt*TOGT1 (AAB36653), *Os*UGT709A4 (BAC80066), *Os*ZOGT1 (BAS90436), *Os*ZOGT3 (BAS90518), *Pg*UGT72BD1 (MN124519), *Pg*UGT73AL1 (XP_031381080), *Pg*UGT76Z1 (XP_031382549), *Pg*UGT84A23 (ANN02875), *Pg*UGT84A24 (ANN02877), *Pg*UGT95B2 (MH507175), *Pj*GAT (AYA60333), *Po*UGT90A7 (EU561019), *Po*UGT95A1 (ACB56927), *Sc*UGT5 (BAJ11653), *Sr*UGT73E1 (AAR06917), *Sr*UGT76G1 (AAR06912), *Sr*UGT85C2 (AAR06916), *Vp*UGT88D8 (BAH47552), *Vp*UGT94F1 (BAI44133), *Vv*GT7 (XP_002276546), *Vv*GT15 (XP_002281513), *Vv*UGT1 (CBI34463), *Vv*UGT95B6 (XP_010664783), *Zm*UFGT1 (P16167), *Zm*UGT74A1 (NP_001105326), *Zm*UGT91L1 (NP_001347041), and *Zm*cisZOG1 (AAK53551). *Ac*, *Allium cepa*; *As*, *Avena strigosa*; *At*, *Arabidopsis thaliana*; *Bv*, *Beta vulgaris*; *Ca*, *Centella asiatica*; *Co*, *Consolida orientalis*; *Cp*, *Citrus paradise*; *Cs*, *Citrus sinensis*; *Ct*, *Clitoria ternatea*; *Gb*, *Ginkgo biloba*; *Ge*, *Glycyrrhiza echinata*; *Gm*, *Glycine max*; *Gt*, *Gentiana triflora*; *Gu*, *Glycyrrhiza uralensis*; *Lg*, *Lamium galeobdolon*; *Me*, *Manihot esculenta*; *Mt*, *Medicago truncatula*; *Nt*, *Nicotiana tabacum*; *Os*, *Oryza sativa*; *Pg*, *Punica granatum*; *Pj*, *Panax japonicus*; *Po*, *Pilosella officinarum*; *Sc*, *Sinningia cardinalis*; *Sr*, *Stevia rebaudiana*; *Vp*, *Veronica persica*; *Vv*, *Vitis vinifera*; *Zm*, *Zea mays*.

## Figures and Tables

**Table 1:** UGTs that showed higher expression in anthers than petals of pomegranate flowers in the transcriptome analysis.

Gene ID	Anther (RPKM)	Petal (RPKM)	Fold Difference	Annotation
*Pgr025859*	46.62	0	-	UDP-glycosyltransferase 76C4 (*Vitis vinifera*)
*Pgr010547*	1529.03	1.66	917.81	Flavonoid 3-*O*-glucosyltransferase (*Medicago truncatula*)
*Pgr007463*	873.01	3.11	280.04	UDP-glycosyltransferase 79B6 (*Arabidopsis thaliana*)
*Pgr004136*	78.25	1.41	55.43	Scopoletin glucosyltransferase (*Nicotiana tabacum*)
*Pgr010383*	562.65	12.11	46.44	UDP-glucose iridoid glucosyltransferase-like (*Eucalyptus grandis*)
*Pgr025860* (*PgUGT76Z1*)	104.82	2.66	39.32	UDP-glycosyltransferase 76F1 (*Arabidopsis thaliana*)
*Pgr020500*	158.14	4.33	36.5	UDP-glycosyltransferase 83A1 (*Arabidopsis thaliana*)
*Pgr003390* (*PgUGT73AL1*)	33,879.19	949.73	35.67	Scopoletin glucosyltransferase-like (*Eucalyptus grandis*)
*Pgr010803*	72.87	2.82	25.82	UDP-glycosyltransferase 74E2-like (*Eucalyptus grandis*)
*Pgr000716*	85.27	10.84	7.86	Zeatin *O*-glucosyltransferase-like (*Jatropha curcas*)

RPKM, reads per kilo bases per million reads.

## Data Availability

The data presented in this study are available in the article.

## Supplementary Materials

The following supporting information can be downloaded at https://www.mdpi.com/article/10.3390/plants11060810/s1, Figure S1: KEGG pathway enrichment analysis of differentially expressed genes (DEGs) in anthers and petals of pomegranate flowers. Table S1: High-resolution electrospray ionization mass spectrometry (HR-ESI-MS) data of metabolites extracted from pomegranate anthers. Rt, retention time; Table S2: High-resolution electrospray ionization mass spectrometry (HR-ESI-MS) data of metabolites extracted from pomegranate petals. Rt, retention time; Table S3: Summary of the RNAseq data; Table S4: RNAseq mapped reads and distributions; Table S5: Genes that are more abundantly expressed in petals relative to anthers of pomegranate flowers; Table S6: Genes that are more abundantly expressed in anthers relative to petals of pomegranate flowers.

## Abbreviations

DEG, differentially expressed gene; HHDP, hexahydroxydiphenoyl; HPLC, high-performance liquid chromatography; HT, hydrolyzable tannin; IPTG, isopropyl β-D-1-thiogalactopyranoside; ISV, ion spray voltage; KEGG, Kyoto Encyclopedia of Genes and Genomes; LC-HR-ESI-MS, liquid chromatography high-resolution electrospray ionization mass spectrometry; MEGA, Molecular Evolutionary Genetics Analysis; MS, mass spectrometry; MUSCLE, Multiple Sequence Comparison by Log-Expectation; NCBI, National Center for Biotechnology Information; NMR, nuclear magnetic resonance; ORF, open reading frame; RPKM, reads per kilo bases per million reads; SRA, sequence reads archive; UGT, UDP-dependent glycosyltransferases; UPLC, ultra-performance liquid chromatography
