# Supplementary material for: Identification and Characterization of Two Regiospecific Tricetin UDP-Dependent Glycosyltransferases from Pomegranate (Punica granatum L.)
Source: Plants (Basel). 2022 Mar 18;11(6):810. doi: 10.3390/plants11060810 (PMC8948884; doi:10.3390/plants11060810)
Supplement: Supplementary file 1 [file plants-11-00810-s001.zip › Table S1-S4.pdf]

Table S1. High-resolution electrospray ionization mass spectrometry (HR-ESI-MS) data of metabolites extracted from pomegranate anthers. Rt, retention time.

Peak	R_t (min)	$[M+NH_4]^+$/$[M+H]^+$/$[M-H]^-$ (*m/z*)	MS/MS (*m/z*)	Calculated formula	Tentative identification
1	0.98	-/-/154.0621	-/-/93.0457, 110.0722, 137.0356	$C_6H_9N_3O_2$	L-histidine
2	1.00	-/-/146.0495	-/-/102.0560, 128.0353	$C_5H_{10}N_2O_3$	Glutamine
3	1.03	-/-/165.0402	-/-/75.0086, 129.0192	$C_6H_6N_4O_2$	7-Methylxanthine
4	1.04	-/-/135.0371	-/-/75.0087, 89.0244	$C_4H_4O_5$	L-Threonic acid
5	1.04	-/-/341.1091	-/-/59.0138, 71.0137, 89.0243, 101.0243, 119.0347, 179.0560, 341.1091	$C_{12}H_{22}O_{11}$	Trehalose
6	1.04	-/-/377.0854	-/-/341.1091	$C_{18}H_{18}O_9$	Unknown
7	1.12	-/-/300.0489	-/-/78.9589, 96.9694	$C_8H_{16}NO_9P$	N-Acetyl-α-D-glucosamine 1-phosphate
8	1.12	-/-/179.0559	-/-/59.0137, 75.0086, 143.0384, 161.0454	$C_6H_{12}O_6$	D-(-)-Fructose
9	1.12, 1.37	-/136.02/134.0142	-/91.05, 119.05, 136.02/71.0138, 89.0243, 115.0036, 133.0142	$C_4H_6O_5$	Malic acid
10	1.14	-/-/149.0453	-/-/59.0138, 75.0086, 131.0348	$C_5H_{10}O_5$	D-(-)-Arabinose
11	1.15	-/-/259.0294	-/-/78.9589, 96.9692, 138.9702, 198.9908	$C_6H_{13}O_9P$	D-Glucose 6-phosphate
12	1.18	-/-/439.0858	-/-/78.9589, 96.9694	$C_{19}H_{20}O_{12}$	Unknown
13	1.38	-/-/323.0283	-/-/78.9590, 96.9694, 111.0200	$C_9H_{13}N_2O_9P$	Uridine monophosphate (UMP)
14	1.43	-/-/188.0564	-/-/98.0246, 114.0559, 142.0508, 144.0665	$C_7H_{11}NO_5$	N-Acetyl-DL-glutamic acid
15	1.54	-/-/128.0352	-/-/128.0352	$C_5H_7NO_3$	4-oxoproline
16	1.55	-/-/191.0197	-/-/85.0294, 87.0086, 111.0086, 129.0193	$C_6H_8O_7$	D-(-)-Quinic acid; Citric acid
17	1.60	-/-/611.1443	-/-/128.0351, 143.0461, 306.0767	$C_{20}H_{32}N_6O_{12}S_2$	L-Glutathione oxidized
18	1.88	350.0997/-/331.0672	109.0287, 127.0389, 145.0495, 153.0179, 171.0285, 315.0710, /-/125.0242, 151.0035, 169.0141, 211.0247, 241.0357, 271.0451	$C_{13}H_{16}O_{10}$	Galloyl glucoside
19	2.25, 7.70, 8.75	652.1132/-/633.0740	259.0233, 277.0338, 303.0129, 447.0553/-/125.0241, 169.0141, 231.0295, 249.0403, 275.0198, 300.9989	$C_{27}H_{22}O_{18}$	6-*O*-galloyl-2, 3-(S)-hexahydroxydiphenoyl -D-glucose; Corilagin; Isocorilagin; Hippomanin A; Gemin D; Strictinin (galloyl

					HHDP glucoside)
20	2.27	-/171.0288/169.0141	-/81.0341, 107.0130, 109.0286, 125.0233, 127.0389, 153.0180/125.0242	$C_7H_6O_5$	Gallic acid
21	2.29	-/-/153.0192	-/-/83.0138, 151.0036	$C_7H_6O_4$	2,4-Dihydroxybenzoic acid
22	2.42, 4.45, 5.89, 7.12	502.1180/-/483.0782	127.0390, 153.0180, 171.0285, 297.0598, 315.0703/-/125.0243, 169.0141, 313.0565, 331.0675	$C_{20}H_{20}O_{14}$	(Digalloyl glucoside)<br>Gallic acid 3-*O*-β-D-(6′-*O*-galloyl)-glucopyranoside
23	2.54	-/345.0812/343.0669	-/153.0180/191.0561	$C_{14}H_{16}O_{10}$	Unknown
24	3.04	-/-/295.1035	-/-/59.0138, 71.0137, 89.0243, 101.0244, 119.0349	$C_{11}H_{20}O_9$	Unknown
25	4.37	-/467.0816/-	-/153.0180/-	$C_{20}H_{18}O_{13}$	Unknown
26	4.44	-/-/218.1033	-/-/88.0403, 146.0822	$C_9H_{17}NO_5$	Pantothenic acid
27	4.65	-/-/325.0565	-/-/93.0343, 125.0242, 169.0141	$C_{14}H_{14}O_9$	Unknown
28	4.79	-/-/487.1098	-/-/125.0242, 169.0141, 211.0249, 331.0672, 469.0991	$C_{20}H_{24}O_{14}$	Galloyl-hexoside derivative
29	5.36	-/-/309.1193	-/-/59.0137, 89.0243, 101.0244, 119.0349	$C_{12}H_{22}O_9$	Unknown
30	6.08	-/-/203.0828	-/-/74.0247, 116.0505, 142.0662, 159.0928	$C_{11}H_{12}N_2O_2$	D-(+)-tryptophan
31	6.26	-/469.1339/467.1198	-/139.0388/125.0243, 137.0247, 167.0349, 179.0347, 219.0655, 305.0666	$C_{21}H_{24}O_{12}$	Unknown
32	6.65	-/595.1439/-	-/271.0595, 433.1123/-	$C_{27}H_{31}O_{15}^+$	Cyanidin-3-*O*-rutinoside
33	6.67	-/497.0923/495.0784	-/153.0180, 305.0285/191.0561, 343.0669	$C_{21}H_{20}O_{14}$	Unknown
34	6.74	-/-/611.1619	-/-/93.0345, 149.0244, 165.0190, 193.0140, 269.0453, 287.0557, 355.0678, 449.1100	$C_{27}H_{31}O_{16}^+$	Cyanidin-3,5-di-*O*-glucoside
35	6.80	-/-/401.1089	-/-/134.0372, 149.0607, 178.0270, 193.0505, 355.1036	$C_{17}H_{22}O_{11}$	Unknown
36	6.81	-/-/355.1035	-/-/134.0372, 149.0606, 178.0270, 193.0505	$C_{16}H_{20}O_9$	Unknown
37	7.00	-/185.0442/183.0296	-/126.0311, 153.0180, 171.0285/124.0164, 168.0063	$C_8H_8O_5$	Unknown
38	7.24, 8.47	804.1237/-/785.0843	153.0181, 259.0233, 277.0338, 303.0131, 467.0816, 617.0771, 787.1013/-/125.0244, 169.0141, 231.0297, 249.0404, 275.0198,	$C_{34}H_{26}O_{22}$	(Digalloyl HHDP glucoside)<br>1,2-di-*O*-galloyl-4,6-*O*-(*S*)-hexahydroxydiphenoylβ-D-glucopyranoside；

			300.9989		Tercatain/1,4-di-*O*-galloyl-3,6-(R)-hexahydroxydiphenyl-β-glucopyranose； TellimagrandinI； PedunculaginII
39	7.51	-/-/323.1346	-/-/59.0138, 71.0137, 89.0243, 101.0243, 113.0244	$C_{13}H_{24}O_9$	Unknown
40	7.59	818.1030/-/799.0637	259.0233, 277.0340, 303.0131, 337.0185, 463.0507, 765.0567, 801.0789/-/300.9989, 479.0468	$C_{34}H_{24}O_{23}$	Granatin A (Ellagic acid derivative)
41	7.60, 8.28, 8.88, 9.15, 9.74	654.1290/-/635.0895	153.0182, 449.0721, 467.0818/-/125.0243, 169.0141, 313.0567, 465.0678, 483.0781	$C_{27}H_{24}O_{18}$	1,2,3-tri-*O*-galloyl-β-glucopyranose; 1,2,4-tri-*O*-galloyl-β-glucopyranose; 1,2,6-tri-*O*-galloyl-β-glucopyranose; 1,3,4-tri-*O*-galloyl-β-glucopyranose; 1,4,6-tri-*O*-galloyl-β-glucopyranose
42	8.09	-/-/387.1664	-/-/163.1127	$C_{18}H_{28}O_9$	Unknown
43	8.29	-/-/455.1772	-/-/101.0607, 125.0244, 163.0611, 307.1036, 409.1716	$C_{18}H_{32}O_{13}$	Unknown
44	8.38	-/-/387.1663	-/-/59.0138, 163.1127	$C_{18}H_{28}O_9$	Unknown
45	8.77	-/-/305.0700	-/-/59.0138, 96.9599, 225.1132	$C_8H_{18}O_{12}$	Unknown
46	8.78	832.1186/-/813.0793	259.0233, 277.0337, 303.0126, 351.0341, 477.0647, 783.0665, 815.0907/-/300.9990, 479.0453	$C_{35}H_{26}O_{23}$	HHDP glucoside derivative
47	8.90	-/-/281.1394	-/-/171.1178, 189.1285, 237.1497	$C_{15}H_{22}O_5$	4-(2,7-Dihydroxy-6-methyl-2-heptanyl)-3-hydroxybenzoic acid
48	9.04	-/-/172.0872	-/-/130.0872	$C_8 H_{15} N O_3$	2-(Acetylamino)hexanoic acid
49	9.42	-/-/631.0581	-/-/125.0242, 169.0142, 217.0146, 245.0089, 273.0044, 300.9990, 445.0399, 463.0523, 613.0485	$C_{27}H_{20}O_{18}$	HHDP glucoside derivative [(-)-vescalin; castalin; terflavin D]
50	9.46	-/-/627.1572	-/-/125.0242, 133.0294, 177.0192, 285.0404, 339.0726, 465.1038	$C_{27}H_{32}O_{17}$	Unknown
51	9.65	954.1190/-/935.0796	153.0180, 259.0235, 277.0338, 303.0129, 447.0533, 767.0712/-/275.0198, 300.9990	$C_{41}H_{28}O_{26}$	Casuarinin
52	9.70, 10.42	-/-/483.0311	-/-/125.0243, 169.0140, 247.0247, 300.9988,	$C_8H_{20}O_{23}$	(Digalloyl glucoside) Gallic acid 3-*O*-β-D-(6′-*O*-

					galloyl)-glucopyranoside
53	9.74	986.1085/-/967.0688	235.0233, 275.0180, 337.0183, 799.0611/-/247.0247, 273.0042, 300.9991, 905.0679, 931.0482, 949.0594	$C_{41}H_{28}O_{28}$	HHDP glucoside derivative
54	9.77	-/627.1542/625.1412	-/303.0494/271.0248, 300.0276	$C_{27}H_{30}O_{17}$	Unknown
55	9.97	-/-/475.0335	-/-/125.0243, 169.0140, 275.0198, 300.9991, 452.0313, 466.0285	$C_{24}H_{12}O_{11}$	HHDP glucoside derivative
56	10.08	970.1134/-/951.0739	277.0338, 303.0130, 337.0183, 783.0665, 953.0862/-/273.0040, 300.9989, 933.0644	$C_{41}H_{28}O_{27}$	Granatin B (galloyl-HHDP-DHHDP-hexoside)
57	10.09	/-/933.0646	/-/273.0041, 300.9990	$C_{41}H_{26}O_{26}$	Galloylpunicalin; Castalagin
58	10.29	1000.1258/-/981.0853	813.0759/-/245.0089, 273.0040, 300.9989, 945.0648 963.0764	$C_{42}H_{30}O_{28}$	HHDP glucoside derivative
59	10.45	820.1175/-/801.0795	109.0286, 153.0180, 445.0387, 615.0610, 633.0706/-/123.0088, 169.0142, 245.0089, 273.0040, 300.9990, 445.0423, 597.0524, 615.0630, 765.0571, 783.0687	$C_{34}H_{26}O_{23}$	Punigluconin
60	10.65	-/956.1342/937.0958	-/153.0181, 277.0339, 303.0130, 599.0656, 769.0869/125.0242, 169.0141, 249.0403, 275.0198, 300.9989	$C_{41}H_{30}O_{26}$	Punicafolin
61	10.66	-/611.1653/609.1475	-/287.0550, 449.1078/271.0250, 300.0276	$C_{27}H_{30}O_{16}$	Rutin
62	10.68 and 12.42	-/-/335.0408	-/-/183.0297	$C_{15}H_{12}O_{9}$	Flurtamone
63	10.77	-/467.0816/465.1039	-/137.0232, 153.0180, 179.0336, 305.0649/109.0292, 125.0242, 133.0294, 177.0192, 193.0141, 285.0408, 303.0510, 355.0668	$C_{21}H_{22}O_{12}$	Unknown
64	10.79	-/449.0719/447.0934	-/287.0544/285.0404	$C_{21}H_{20}O_{11}$	Flavonoid glycoside
65	10.85	-/-/300.9989	-/-/300.9990	$C_{14}H_{6}O_{8}$	Ellagic acid
66	10.88	1122.1238/-/1103.0846	277.0337, 303.0128, 337.0184, 481.0630, 783.0660, 935.0693/-/273.0041, 300.9989, 933.0639, 951.0739	$C_{48}H_{32}O_{31}$	HHDP glucoside derivative

67	10.98	806.1392/-/787.0999	153.0180, 237.0388, 449.0707, 619.0920/-/125.0243, 169.0141, 313.0566, 465.0674, 617.0777	$C_{34}H_{28}O_{22}$	1,2,4,6-tetra-*O*-galloyl-β-D-glucose
68	11.02	-/-/815.0586	-/-/123.0085, 169.0141, 217.0138, 245.0083, 273.0036, 300.9990. 615.0657, 765.0623, 783.0698	$C_{35}H_{28}O_{23}$	Pedunculagin I derivative (bis-HHDP glucoside derivative)
69	11.24	-/483.1128/481.0989	-/127.0390, 153.0180, 195.0286, 312.0600/125.0243, 149.0243, 193.0142, 257.0455, 355.0670, 463.0889	$C_{21}H_{22}O_{13}$	Unknown
70	11.64	-/449.0719/447.0934	-/287.0544/285.0404	$C_{21}H_{20}O_{11}$	Cynaroside (Luteolin 7-*O*-glycoside);<br>Kaempferol 4'-*O*-glucoside
71	11.68	984.1292/-/965.0900	277.0338, 303.0130, 351.0338, 797.0815/-/245.0089, 273.0042, 300.9991	$C_{42}H_{30}O_{27}$	Pedunculagin I derivative (Bis-HHDP glucoside derivative)
72	11.81	-/451.1001/449.1093	-/121.0285, 127.0389, 153.0180, 163.0387, 289.0700/93.0345, 117.0345, 125.0242, 149.0242, 167.0348, 193.0142, 269.0454, 287.0540, 313.0574, 355.0666	$C_{21}H_{22}O_{11}$	Hovetrichoside C
73	11.91	972.1294/-/953.0890	153.0180, 785.0811/-/125.0243, 169.0141, 217.0142, 245.0092, 273.0041, 300.9990, 617.0771, 767.0731, 917.0720, 935.0803	$C_{41}H_{30}O_{27}$	Galloyl-bis-HHDP-hexoside
74	12.28	958.1503/-/939.1109	153.0181, 771.1024/-/125.0242, 169.0141, 465.0683, 769.0901	$C_{41}H_{32}O_{26}$	1,2,3,4,6-penta-*O*-galloyl-β-D-glucose
75	12.61	1108.1454/-/1089.1057	153.0180, 303.0125, 599.0649, 769.0908, 921.0995/-/125.0139, 169.0139, 275.0200, 300.9990, 937.0954	$C_{48}H_{34}O_{30}$	HHDP glucoside derivative
76	12.90	-/305.0650/303.0511	-/137.0232, 153.0180, 179.0335/109.0293, 125.0242, 133.0294, 151.0399, 177.0191, 193.0142	$C_{15}H_{12}O_7$	Unknown
77	13.04	-/273.0754/271.0147	-/147.0439, 153.0180, 171.0285/119.0501, 151.0037, 177.0193	$C_{15}H_{12}O_5$	Naringenin
78	13.05	-/449.0719/447.0934	-/287.0544/285.0404	$C_{21}H_{20}O_{11}$	Kaempferol 7-*O*-glucoside
79	13.11	-	-/-/151.0035, 271.0613,	$C_{20}H_{18}O_{11}$	Unknown

		/435.0918/433.0780	301.0353		
80	13.32, 13.59	-/-/461.1093	-/-/255.0295, 283.0247, 298.0479, 313.0345, 446.0860	$C_{22}H_{22}O_{11}$	Unknown
81	13.50	-/451.1001/449.1094	-/121.0286, 127.0392, 153.0182, 163.0389, 289.0702/135.0451, 151.0036, 287.0562	$C_{21}H_{22}O_{11}$	Hovetrichoside C
82	13.71	-/449.0719/447.0934	-/287.0544/285.0404	$C_{21}H_{20}O_{11}$	Flavonoid glycosides
83	13.73	-/465.1020/463.0885	-/303.0497/301.0354	$C_{21}H_{20}O_{12}$	Tricetin 4'-*O*-β-glucopyranoside
84	13.82	-/303.0494/301.0352	-/303.0497/301.0354	$C_{15}H_{10}O_7$	Tricetin
85	13.96	-/317.0652/315.0512	-/302.0421/300.0276	$C_{16}H_{12}O_7$	Isorhamnetin
86	13.99	-/479.1178/477.1039	-/317.0053/300.0275, 315.0511	$C_{21}H_{22}O_{12}$	6-*O*-[(2E)-3-(4-Hydroxyphenyl)-2-propenoyl]-1-*O*-(3,4,5-trihydroxybenzoyl)hexopyranose
87	14.66	-/-/487.0522	-/-/183.0297, 335.0410	$C_{22}H_{16}O_{13}$	Unknown
88	14.71	-/289.0701/287.0562	-/121.0285, 153.0181, 163.0388/93.0344, 117.0345, 125.0243, 193.0141	$C_{15}H_{12}O_6$	Eriodictyol
89	14.73	-/-/125.0243	-/-/57.0345	$C_6H_6O_3$	Phloroglucinol
90	14.75	-/-/193.0141	-/-/149.0243, 151.0035	$C_9H_6O_5$	Unknown
91	14.83	-/419.0967/417.0830	-/287.0543/285.0404	$C_{20}H_{18}O_{10}$	Luteolin 3'-*O*-β-xylopyranoside
92	15.30	-/-/263.1288	-/-/151.0764, 201.1280, 204.1155, 219.1390	$C_{15}H_{20}O_4$	Abscisic acid
93	15.80	-/287.0545/285.0405	-/287.0544/285.0406	$C_{15}H_{10}O_6$	Luteolin
94	15.98	-/317.0650/315.0511	-/302.0415/272.0329, 300.0275	$C_{16}H_{12}O_7$	Isorhamnetin
95	17.80	-/271.0597/269.0	-/271.0595/269.0456	$C_{15}H_{10}O_5$	Apigenin

		456			
96	18.20	-/275.0910/273.0769	-/107.0494/119.0499, 123.0451, 167.0348	$C_{15}H_{14}O_5$	Phloretin
97	18.30	-/301.0701/299.0561	-/286.0465/256.0377, 284.0327	$C_{16}H_{12}O_6$	Unknown
98	18.71	-/331.0805/327.2178	-/315.0495, 331.0809/171.1026, 211.1339, 229.1444	$C_{18}H_{32}O_5$	Corchorifatty acid F
99	19.74	-/-/457.1720	-/-/125.0244, 169.0142	$C_{21}H_{30}O_{11}$	Unknown
100	19.88	-/-/401.0878	-/-/121.0294, 225.0558, 313.0725	$C_{20}H_{18}O_9$	Unknown
101	19.95	-/-/329.2334	-/-/171.1026, 211.1340, 229.1444	$C_{18}H_{34}O_5$	(15Z)-9,12,13-Trihydroxy-15-octadecenoic acid
102	21.49	-/-/643.2777	-/-/136.0767, 190.0507, 339.1352, 611.2536	$C_{34}H_{44}O_{12}$	Unknown
103	21.68	-/-/293.1759	-/-/221.1546, 236.1053	$C_{17}H_{26}O_4$	Unknown
104	21.70	-/489.2850/487.3434	-/147.0441/487.3434	$C_{30}H_{48}O_5$	Asiatic acid
105	21.86	-/-/309.1709	-/-/162.1049, 219.1753, 281.1758	$C_{17}H_{26}O_5$	Unknown
106	21.92	-/-/239.1289	-/-/154.0635, 167.1440, 195.1390	$C_{13}H_{20}O_4$	Unknown
107	22.13	-/-/473.3639	-/-/473.3638	$C_{30}H_{50}O_4$	Punicanolic acid
108	22.62	-/-/471.3483	-/-/471.3481	$C_{30}H_{48}O_4$	Unknown
109	22.71	-/279.0930/277.1444	-/149.0232/121.0293, 127.1127, 134.0372	$C_{16}H_{22}O_4$	Mono(2-ethylhexyl) phthalate
110	23.09	-/-/452.2784	-/-/255.2330	$C_{21}H_{44}NO_7P$	Glycerophospho-N-palmitoyl ethanolamine
111	23.05	-/-/265.1479	-/-/96.9600	$C_{12}H_{26}O_4S$	Dodecyl sulfate
112	23.35	-/-/347.1714	-/-/217.0713, 301.1661	$C_{16}H_{28}O_8$	Unknown
113	23.94	-/457.3675/455.3532	-/95.0800, 189.1637, 261.1845, 393.3515, 411.3629, 439.3566/455.3533	$C_{30}H_{48}O_3$	Ursolic acid; Oleanolic acid; Hydroxyurs-12-en-23-oic acid
114	24.11	-/-/339.2331	-/-/163.1129	$C_{23}H_{32}O_2$	2,2'-methylenebis(4-methyl-6-tert-butylphenol)
115	24.37	/-/271.2279	/-/225.2223	$C_{16}H_{32}O_3$	16-hydroxyhexadecanoic acid

Table S2. High-resolution electrospray ionization mass spectrometry (HR-ESI-MS) data of metabolites extracted from pomegranate petals. Rt, retention time.

Peak	R_t (min)	$[M+NH_4]^+/[M+H]^+/[M-H]^-$ (*m/z*)	MS/MS (*m/z*)	Calculated formula	Tentative identification
1	1.01	-/-/181.0715	-/-/59.0137, 71.0137, 89.0243, 101.0242, 119.0347, 131.0349, 163.0610, 181.0716	$C_6H_{14}O_6$	D-(-)-mannitol
2	1.03	-/-/135.0297	-/-/75.0087, 89.0244	$C_4H_8O_5$	L-Threonic acid
3	1.03	-/-/146.0458	-/-/102.059, 128.0352	$C_5 H_9 N O_4$	L-Glutamic acid
4	1.04	-/-/341.1091	-/-/59.0138, 71.0137, 89.0243, 101.0243, 119.0347, 179.0560, 341.1091	$C_{12}H_{22}O_{11}$	Trehalose
5	1.04	-/-/165.0402	-/-/75.0086, 129.0192	$C_6H_6N_4O_2$	7-Methylxanthine
6	1.04	-/381.0788/-	-/381.0784/-	$C_{17}H_{16}O_{10}$	Unknown
7	1.06	-/-/377.0853	-/-/341.1091	$C_{18}H_{18}O_9$	Unknown
8	1.06	-/-/179.0559	-/-/59.0138, 75.0086, 143.0349, 161.0454	$C_6H_{12}O_6$	D-(-)-Fructose
9	1.09	-/-/149.0453	-/-/59.0138, 75.0086, 131.0348	$C_5H_{10}O_5$	D-(-)-Arabinose
10	1.14	-/-/259.0294	/-/78.9589, 96.9692, 138.9702, 198.9908	$C_6H_{13}O_9P$	D-Glucose 6-phosphate
11	1.23	-/-/439.0858	-/-/78.9589, 96.9694	$C_{19}H_{20}O_{12}$	Unknown
12	1.30	-/136.02/134.0142	-/91.05, 119.05, 136.02/71.0138, 89.0243, 115.0036, 133.0142	$C_4H_6O_5$	Malic acid
13	1.53	-/-/481.0627	-/-/275.0198, 300.9989	$C_{20}H_{18}O_{14}$	HHDP glucoside derivative
14	1.58	-/-/191.0197	-/-/85.0294, 87.0086, 111.0086, 129.0193	$C_6H_8O_7$	D-(-)-Quinic acid ; Citric acid
15	1.71	-/-/645.1889	-/-/71.0137, 113.0243	$C_{24}H_{38}O_{20}$	Unknown
16	1.81, 7.12	-/315.0710/-	-/153.0180/-	$C_{13}H_{14}O_9$	Unknown
17	1.87	350.0997/-/331.0670	109.0287, 127.0389, 145.0495, 153.0179, 171.0285, 315.0710/-/125.0242, 151.0036, 169.0140, 211.0246, 271.0454	$C_{13}H_{16}O_{10}$	Galloyl glucoside
18	2.26	-/171.0288/169.0141	-/81.0341, 107.0130, 109.0286, 125.0233, 127.0389, 153.0180/125.0242	$C_7H_6O_5$	Gallic acid
19	2.27	-/-/777.2307	-/-/113.0242	$C_{29}H_{46}O_{24}$	Unknown
20	2.57	-/345.0814/343.0670	-/153.0180/191.0560	$C_{14}H_{16}O_{10}$	Unknown
21	4.45	-/-/218.1033	-/-/88.0403, 146.0822	$C_9H_{17}NO_5$	Pantothenic acid
22	4.46	-/-/325.0565	-/-/93.0343, 125.0242, 169.0141	$C_{14}H_{14}O_9$	Unknown
23	4.79	-/-/487.1096	-/-/125.0242, 169.0140, 211.0250,	$C_{20}H_{24}O_{14}$	Galloyl-hexoside derivative

			331.0663, 469.0993		
24	4.99	-/-/299.0771	-/-/59.0137, 71.0137, 89.0242, 101.0242, 119.0349, 137.0243, 179.0348, 239.0564	$C_{13}H_{16}O_8$	Unknown
25	5.45	-/-/315.0722	-/-/125.0238, 151.0037, 169.0138	$C_{13}H_{16}O_9$	Galloyl-hexoside derivative
26	6.54	-/-/315.0511	-/-/153.0192	$C_{13}H_{16}O_9$	Unknown
27	6.62	-/595.1650/-	-/271.0594, 433.1121/-	$C_{27}H_{31}O_{15}^+$	Cyanidin-3-*O*-rutinoside
28	6.63	-/-/611.1616	-/-/93.0344, 149.0242, 165.0191, 193.0141, 269.0456, 287.0561, 355.0671, 449.1091	$C_{27}H_{31}O_{16}^+$	Cyanidin-3,5-di-*O*-glucoside
29	6.99	-/185.0442/183.0297	-/126.0311, 153.0180, 171.0285/124.0164, 168.0063	$C_8H_6O_5$	Unknown
30	7.01	-/-/957.0849	-/-/275.0198, 300.9989	$C_{40}H_{30}O_{28}$	HHDP glucoside derivative
31	7.09	-/-/483.0782	-/-/125.0243, 169.0141, 211.0247, 271.0459, 313.0561, 331.0685	$C_{20}H_{20}O_{14}$	Galloyl-hexoside derivative
32	7.30, 8.53	804.1237/-/785.0848	153.0181, 259.0233, 277.0338, 303.0131, 467.0816, 617.0771, 787.1013/-/125.0242, 169.0140, 231.0297, 249.0403, 275.0198, 300.9989	$C_{34}H_{26}O_{22}$	1,2-di-*O*-galloyl-4,6-*O*-(*S*)-hexahydroxydiphenoylβ-D-glucopyranoside；Tercatain/1,4-di-*O*-galloyl-3,6-(R)-hexahydroxydiphenyl-β-glucopyranose；TellimagrandinI；PedunculaginII
33	7.62	818.1035/-/799.0640	259.0233, 277.0340, 303.0131, 337.0185, 463.0507, 765.0567, 801.0789/-/300.9989, 479.0467	$C_{34}H_{24}O_{23}$	Granatin A (Ellagic acid derivative)
34	7.67	652.1132/-/633.0738	259.0233, 277.0338, 303.0129, 447.0553/-/169.0140, 249.0404, 275.0195, 300.9988	$C_{27}H_{22}O_{18}$	6-*O*-galloyl-2, 3-(S) -hexahydroxydiphenoyl -D-glucose; Corilagin; Isocorilagin; Hippomanin A; Gemin D; Strictinin (galloyl HHDP glucoside)
35	8.27, 8.87, 9.69	654.1293/-/635.0891	-153.0182, 449.0721, 467.0818/-/125.0242, 169.0141, 313.0566, 465.0673	$C_{27}H_{24}O_{18}$	1,2,3-tri-*O*-galloyl-β-glucopyranose; 1,2,4-tri-*O*-galloyl-β-glucopyranose; 1,2,6-tri-*O*-galloyl-β-glucopyranose; 1,3,4-tri-*O*-galloyl-β-glucopyranose; 1,4,6-tri-*O*-galloyl-β-glucopyranose
36	8.48,	-/433.1127/-	-/271.0594/-	$C_{21}H_{20}O_{10}$	Unknown

	13.07				
37	8.73	832.1191/-/813.0797	259.0233, 277.0337, 303.0126, 351.0341, 477.0647, 783.0665, 815.0907/-/300.9989, 479.0470	$C_{35}H_{26}O_{23}$	HHDP glucoside derivative
38	8.83	-/-/305.0700	-/-/59.0138, 96.9599, 225.1132	$C_{8}H_{18}O_{12}$	Unknown
39	8.89, 9.70	-/467.0819/-	-/153.0179/-	$C_{20}H_{18}O_{13}$	Unknown
40	9.11	-/451.1225/449.1093	-/121.0284, 289.0699/125.0242, 259.0610, 287.0562, 421.1139	$C_{21}H_{22}O_{11}$	Hovetrichoside C
41	9.26	-/-/329.0878	-/-/123.0450, 167.0348	$C_{14}H_{18}O_{9}$	Unknown
42	9.43	-/-/631.0580	-/-/125.0242, 169.0141, 217.0139, 245.0090, 273.0041, 300.9988, 445.0415, 463.0515, 613.0463	$C_{27}H_{20}O_{18}$	HHDP glucoside derivative [(-)-vescalin; castalin; terflavin D]
43	9.47	-/449.0719/447.0933	-/287.0544/165.0191, 285.0404, 327.0722	$C_{21}H_{20}O_{11}$	Flavonoid glycosides
44	9.53	646.1968/-/627.1567	95.0495, 121.0284, 139.0388, 167.0337, 305.0648/-/139.0399, 165.0191, 345.0826, 447.0933	$C_{27}H_{32}O_{17}$	Unknown
45	9.71, 10.39	986.1091/-/967.0685	235.0233, 275.0180, 337.0183, 799.0611/-/247.0246, 273.0040, 300.9989, 905.0681, 931.0482, 949.0578	$C_{41}H_{28}O_{28}$	HHDP glucoside derivative
46	9.79	-/611.1653/609.1475	-/287.0550, 449.1078/285.0403, 447.0932	$C_{27}H_{30}O_{16}$	Rutin
47	10.14	820.1189/-/801.0789	109.0286, 153.0180, 445.0387, 615.0610, 633.0706/-/123.0086, 169.0142, 245.0089, 273.0039, 300.9988, 445.0423, 597.0524, 615.0630, 765.0580, 783.0685	$C_{34}H_{26}O_{23}$	Punigluconin
48	10.15	970.1136/-/951.0737	277.0338, 303.0130, 337.0183, 783.0665, 953.0862/-/273.0040, 300.9988, 933.0644	$C_{41}H_{28}O_{27}$	Granatin B (galloyl-HHDP-DHHDP-hexoside)
49	10.37, 12.28	1000.1258/-/981.0852	813.0759/-/245.0089, 273.0040, 300.9988, 945.0665, 963.0736	$C_{42}H_{30}O_{28}$	HHDP glucoside derivative
50	10.55, 11.14	1144.1307/-/1125.0906	109.0286, 153.0181, 221.0075, 337.0183, 449.0366, 957.0822/-/247.0246, 273.0041, 300.9988, 907.0856, 933.0618, 949.0560, 1081.0951	$C_{47}H_{34}O_{33}$	HHDP glucoside derivative
51	10.78	1128.1356/-	221.0076, 303.0128, 941.0868/-	$C_{47}H_{34}O_{32}$	bis-HHDP glucoside

		/1109.0951	/275.0197, 300.9988, 633.0724, 935.0798		derivative
52	10.97	-/-/497.3349	-/-/433.3182, 451.3292	$C_{24}H_{50}O_{10}$	Unknown
53	11.00	-/453.3429/-	-/100.1124, 210.1485, 435.3331/-	$C_{23}H_{48}O_8$	Unknown
54	11.08	834.1350/-/815.0955	81.0341, 109.0286, 153.0179, 445.0390, 615.0604, 647.0864/-/123.0085, 169.0141, 217.0138, 245.0089, 273.0042, 300.9990. 615.0608, 765.0570, 783.0682	$C_{35}H_{28}O_{23}$	Pedunculagin I derivative (bis-HHDP glucoside derivative)
55	11.21	806.1392/-/787.1005	153.0180, 237.0388, 449.0707, 619.0920/-/125.0243, 169.0141, 313.0580, 465.0677, 617.0788	$C_{34}H_{28}O_{22}$	1,2,4,6-tetra-*O*-galloyl-β-D-glucose
56	11.62	984.1292/-/965.0898	277.0338, 303.0130, 351.0338, 797.0815/-/245.0089, 273.0041, 300.9989	$C_{42}H_{30}O_{27}$	Pedunculagin I derivative (Bis-HHDP glucoside derivative)
57	11.89	972.1292/-/953.0889	153.0180, 785.0811/-/125.0243, 169.0141, 217.0142, 245.0092, 273.0041, 300.9990, 617.0811, 767.0731, 917.0720, 935.0793	$C_{41}H_{30}O_{27}$	Galloyl-bis-HHDP-hexoside
58	12.06, 12.64	680.1086/-/661.0690	69.0342, 127.0390, 153.0178, 245.0071, 305.0286, 331.0076, 349.0176, 475.0492, 493.0602/-/217.0146, 303.0143, 491.0478	$C_{28}H_{22}O_{19}$	Unknown
59	12.24	-/-/441.1980	-/-/101.0243, 263.1496, 395.1918	$C_{18}H_{34}O_{12}$	Unknown
60	12.25	-/-/395.1923	-/-/101.0243, 113.0244, 161.0452, 263.1502	$C_{17}H_{32}O_{10}$	Unknown
61	12.55	-/449.1072/447.0936	-/287.0544/227.0349, 255.0297, 284.0326, 285.0401	$C_{21}H_{20}O_{11}$	Astragaline (Kaempferol -3-*O*-glucoside)
62	13.14	-/433.1127/431.0985	-/271.0595/268.0376, 269.0454	$C_{21}H_{20}O_{10}$	Apigenin 4'-*O*-β-glucopyranoside
63	13.88	-/-/455.2136	-/-/71.0137, 101.0243, 277.1653,409.2078	$C_{19}H_{36}O_{12}$	Unknown
64	14.73	-/289.0701/287.0561	-/121.0285, 153.0181, 163.0388/93.0344, 117.0342, 125.0243, 167.0351, 193.0141	$C_{15}H_{12}O_6$	Eriodictyol
65	15.02	-/-/435.1299	-/-/167.0348, 273.0769, 297.0772	$C_{21}H_{24}O_{10}$	Phlorizin
66	15.11	-/-/515.1772	-/-/125.0241, 169.0140, 201.1132,225.1135, 243.1235, 363.1662	$C_{23}H_{32}O_{13}$	Unknown
67	16.18	-/-/569.3182	-/-/287.2227, 449.2758, 523.3090	$C_{26}H_{50}O_{13}$	Unknown
68	18.74	-/-/615.2659	-/-/125.0241, 169.0137, 283.1910,	$C_{29}H_{44}O_{14}$	Unknown

			301.2025		
69	19.87	-/-/401.0876	-/-/121.0293, 225.0553, 313.0717	$C_{20}H_{18}O_9$	Unknown
70	19.98	-/-/357.0616	-/-/121.0293, 357.0617	$C_{18}H_{14}O_8$	Unknown
71	21.40	-/-/503.3378	-/-/503.3378	$C_{30}H_{48}O_6$	Arjungenin
72	21.50	-/-/549.3436	-/-/503.3377	$C_{31}H_{50}O_8$	Unknown
73	21.72	-/489.2850/487.3427	-/147.0441/487.3430	$C_{30}H_{48}O_5$	Asiatic acid
74	21.82	-/-/309.1707	-/-/162.1049, 219.1753, 281.1758	$C_{17}H_{26}O_5$	Unknown
75	21.90	-/-/239.1289	-/-/154.0635, 167.1440, 195.1390	$C_{13}H_{20}O_4$	Unknown
76	22.13	-/-/473.3637	-/-/473.3638	$C_{30}H_{50}O_4$	Punicanolic acid
77	22.29	-/-/485.3275	-/-/455.3180	$C_{30}H_{46}O_5$	Unknown
78	22.64	-/-/471.3482	-/-/471.3481	$C_{30}H_{48}O_4$	Unknown
79	22.70	-/279.0930/277.1444	-/149.0232/121.0293, 127.1127, 134.0372	$C_{16}H_{22}O_4$	Mono(2-ethylhexyl) phthalate
80	23.09	-/-/452.2784	-/-/255.2330	$C_{21}H_{44}NO_7P$	Glycerophospho-N-palmitoyl ethanolamine
81	23.31	-/-/265.1479	-/-/96.9600	$C_{12}H_{26}O_4S$	Dodecyl sulfate
82	23.36	-/-/347.1713	-/-/217.0713, 301.1661	$C_{16}H_{28}O_8$	Unknown
83	23.94	-/-/455.3530	-/-/455.3532	$C_{30}H_{48}O_3$	Ursolic acid; Oleanolic acid
84	24.11	-/-/339.2325	-/-/163.1129	$C_{23}H_{32}O_2$	2,2'-methylenebis(4-methyl-6-tert-butylphenol
85	25.17	-/-/325.1842	-/-/183.0118	$C_{18}H_{30}O_3S$	4-Dodecylbenzenesulfonic acid

Table S3. Summary of RNAseq data.

Sample	Raw reads	Raw bases (bp)	Clean reads	Clean bases (bp)	Error rate (%)	Q20 (%)	Q30 (%)
Pg_anther2	42350106	6394866006	42102446	6337271438	0.00208	97.07	92.62
Pg_anther4	41708934	6298049034	41225410	6212382316	0.008355	95.08	88.76
Pg_anther9	45340278	6846381978	44964900	6773780416	0.008987	95.62	89.75
Pg_petal2	44916484	6782389084	44659014	6722178230	0.002313	97.04	92.72
Pg_petal4	46465480	7016287480	45870616	6910089572	0.00677	95.22	89
Pg_petal9	42646274	6439587374	42198864	6355860646	0.007268	95.61	89.71

Table S4. RNAseq mapped reads and distributions.

Sample	Total mapped	Multiple mapped	Uniquely mapped	Gene	Exon	Intergenic
Pg_anther2	39009104 (92.65%)	1931146 (4.95%)	37077958 (95.05%)	32475642 (87.59%)	31859470 (98.10%)	4602316 (12.41%)
Pg_anther4	36046221 (87.44%)	2695755 (7.48%)	33350466 (92.52%)	28592716 (85.73%)	28135489 (98.40%)	4757750 (14.27%)
Pg_anther9	39758843 (88.42%)	2133951 (5.37%)	37624892 (94.63%)	32581483 (86.60%)	31490897 (96.65%)	5043409 (13.40%)
Pg_petal2	41098186 (92.03%)	3642607 (8.86%)	37455579 (91.14%)	31020157 (82.82%)	30016225 (96.76%)	6435422 (17.18%)
Pg_petal4	40602543 (88.52%)	2110683 (5.20%)	38491860 (94.80%)	33369330 (86.69%)	32170512 (96.41%)	5122530 (13.31%)
Pg_petal9	37710951 (89.36%)	2682376 (7.11%)	35028575 (92.89%)	29780137 (85.02%)	28765272 (96.59%)	5248438 (14.98%)
