# Supplementary figures and images for: Identification and Characterization of Two Regiospecific Tricetin UDP-Dependent Glycosyltransferases from Pomegranate (Punica granatum L.)
Source: Plants (Basel). 2022 Mar 18;11(6):810. doi: 10.3390/plants11060810 (PMC8948884; doi:10.3390/plants11060810)

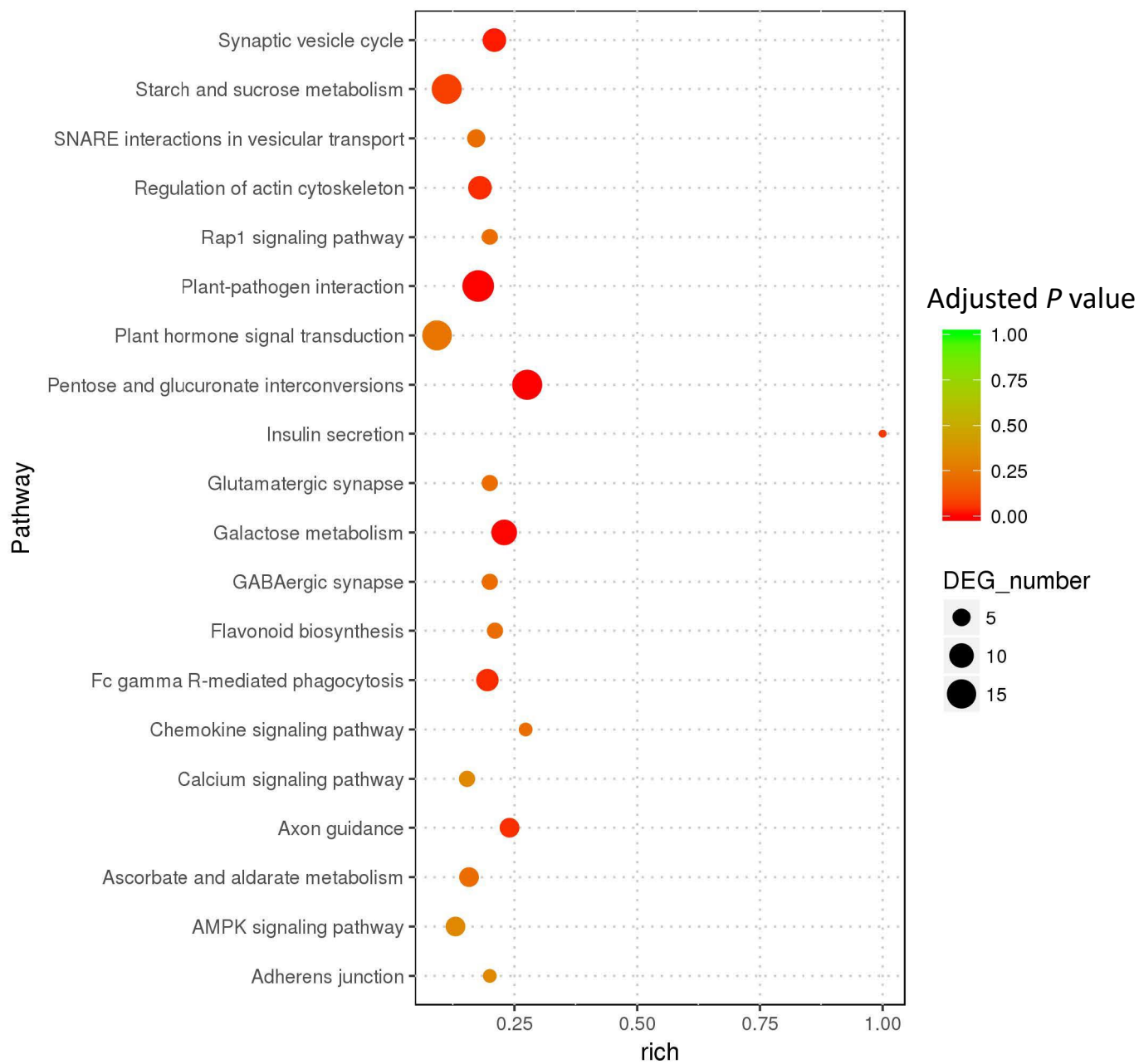

Supplement: Supplementary file 1 [file plants-11-00810-s001.zip › Figure S1.pdf]
